# Electroacupuncture Pretreatment Attenuates Cerebral Ischemic Injury via Notch Pathway-Mediated Up-Regulation of Hypoxia Inducible Factor-1α in Rats

**DOI:** 10.1007/s10571-015-0203-9

**Published:** 2015-05-15

**Authors:** Yu Zhao, Bin Deng, Yichong Li, Lihua Zhou, Lei Yang, Xingchun Gou, Qiang Wang, Guozhong Chen, Hao Xu, Lixian Xu

**Affiliations:** 1 State Key Laboratory of Military Stomatology, Department of Anesthesiology, School of Stomatology, The Fourth Military Medical University, Xi’an, 710032 China; 2 Department of Anesthesiology, Binghua Hospital, Haerbin, 150080 China; 3 Institution of Basic Medical Science, Xi’an Medical University, Xi’an, 710032 China; 4 Department of Anesthesiology, Xijing Hospital, Fourth Military Medical University, Xi’an, 710032 China; 5 Department of Anesthesiology, Fuzhou General Hospital, Fuzhou, 350015 China

**Keywords:** HIF-1α, Notch pathway, Stroke, Penumbra, Electroacupuncture, Neurobehavior function, Apoptosis

## Abstract

We have reported electroacupuncture (EA) pretreatment induced the tolerance against focal cerebral ischemia through activation of canonical Notch pathway. However, the underlying mechanisms have not been fully understood. Evidences suggest that up-regulation of hypoxia inducible factor-1α (HIF-1α) contributes to neuroprotection against ischemia which could interact with Notch signaling pathway in this process. Therefore, the current study is to test that up-regulation of HIF-1α associated with Notch pathway contributes to the neuroprotection of EA pretreatment. Sprague–Dawley rats were treated with EA at the acupoint “Baihui (GV 20)” 30 min per day for successive 5 days before MCAO. HIF-1α levels were measured before and after reperfusion. Then, HIF-1α antagonist 2ME2 and γ-secretase inhibitor MW167 were used. Neurologic deficit scores, infarction volumes, neuronal apoptosis, and Bcl2/Bax were evaluated. HIF-1α and Notch1 intracellular domain (NICD) were assessed. The results showed EA pretreatment enhanced the neuronal expression of HIF-1α, reduced infarct volume, improved neurological outcome, inhibited neuronal apoptosis, up-regulated expression of Bcl-2, and down-regulated expression of Bax after reperfusion in the penumbra, while the beneficial effects were attenuated by 2ME2. Furthermore, intraventricular injection with MW167 efficiently suppressed both up-regulation of NICD and HIF-1α after reperfusion. However, administration with 2ME2 could only decrease the expression of HIF-1α in the penumbra. In conclusion, EA pretreatment exerts neuroprotection against ischemic injury through Notch pathway-mediated up-regulation of HIF-1α.

## Introduction

Stroke is a major cause of death and disability in adults throughout the world, and only a minority of stroke patients receives thrombolytic therapy due to the narrow time window and side effects associated with the thrombolytic agent (Go et al. 2013). Therefore, new strategies focusing on neuroprotection are urgently needed. Preconditioning, as a potent endogenous protective procedure, activates several endogenous signaling pathways that protect against ischemia (Wang et al. 2009). Electroacupuncture (EA) has been shown to produce clinically beneficial effects in stroke patients, and EA pretreatment can also induce tolerance against ischemia (Zhao et al. 2012). However, the signaling mechanisms mediating the effects of EA pretreatment are unclear.

HIF-1α is a transcription factor that plays a key role in regulating the adaptive response to hypoxia by regulating the expression of its target genes, which include heme oxygenase-1 (HO-1) (Singh et al. 2012). HIF-1α has been shown to protect neurons from death during the initial 24 h following mild hypoxia (Lopez-Hernandez et al. 2012). Previous studies have shown that the neuroprotection against focal cerebral ischemia provided by various agents is through the up-regulation of HIF-1α (Wang et al. 2012a; Yuan et al. 2011; Doeppner et al. 2012). Whether HIF-1α acts in a similar manner after EA neuroprotective pretreatment is unclear.

Our previous study demonstrated that EA pretreatment-induced tolerance against cerebral ischemia by activating the canonical Notch signaling pathway. A potential link between HIF-1α and Notch signaling has been reported (Wilkins et al. 2009). Additionally, the expression of HIF-1α and its downstream target genes requires the phosphorylation of signal transducer and activator of transcription 3 (STAT3), and Notch signaling can activate STAT3 phosphorylation (Jung et al. 2005; Kamakura et al. 2004). Notch and HIF-1α appear to share several levels of molecular regulatory mechanisms.

Therefore, the aims of the current study are to test the hypothesis that the up-regulation of HIF-1α contributes to the neuroprotective effects of EA pretreatment and to determine whether the neuroprotection induced by the up-regulation of HIF-1α is associated with the Notch signaling pathway.

## Materials and Methods

### Experimental Protocols

#### Experiment I

To assess the effect of EA pretreatment on the expression of HIF-1α and HO-1 after ischemia, the rats were randomly divided into two groups: Ischemia and reperfusion (I/R) and EA + I/R. The animals were subjected to middle cerebral arterial occlusion (MCAO). The rats in the EA + I/R group were given 30 min of EA stimulation per day for 5 days. At 24 h after the last EA pretreatment and at 2, 6, 24, 48, and 72 h after reperfusion, RT-PCR and Western blot analysis were performed (*n* = 8). At 24 h after reperfusion, HIF-1α/NeuN double immunofluorescence analyses were performed in the Sham, I/R, and EA + I/R groups (*n* = 8).

#### Experiment II

To determine the effects of the HIF-1α inhibitor 2ME2 on the neuroprotection associated with EA pretreatment, rats were randomly divided into five groups: Sham, I/R, EA + I/R, 2ME2 + I/R, EA + 2ME2 + I/R. 2ME2 was administered by intraperitoneal (i.p.) injection at 30 min before MCAO in the 2ME2 + I/R and EA + 2ME2 + I/R groups. At 72 h after reperfusion, neurobehavioral evaluation and infarct assessment were then performed (*n* = 8). At 24 h after reperfusion, TUNEL staining and both Bcl2 and Bax expressions were tested to measure neuronal apoptosis (*n* = 8).

#### Experiment III

To evaluate the interaction between the Notch signaling pathway and HIF-1α levels induced by EA pretreatment, rats were randomly assigned to four groups (*n* = 8 in each group): I/R, EA + I/R, EA + MW167 + I/R (γ-secretase inhibitor) and EA + 2ME2 + I/R. MW167 was administered by intracerebroventricular (i.c.v.) injection 30 min before MCAO in the EA + MW167 + I/R group, and 2ME2 was administered by i.p. injection 30 min before MCAO in the EA + 2ME2 + I/R group. At 24 h after reperfusion, Western blot analysis and immunofluorescence were performed (*n* = 8).

### Electroacupuncture Pretreatment

The EA pretreatment was performed as described previously (Wang et al. 2009). The “Baihui (GV 20)” acupoint was used, which, in rats, is located at the intersection of the sagittal midline and the line linking the two ears. The animals were anesthetized using 40 mg/kg sodium pentobarbital (SP) (i.p.), and then the acupoint “Baihui (GV 20) was stimulated at the intensity of 1 mA and frequency of 2/15 Hz for 30 min per day for five successive days using the G6805–2 EA Instrument (Model No. 227 033; Qingdao Xinsheng Ltd).

### Drug administration

In experiment II, 2ME2 was dissolved in PBS with 10 % dimethyl sulfoxide (DMSO) and administered by i.p. injection 30 min before MCAO in the 2ME2 + I/R and EA + 2ME2 + I/R groups. The same volume of vehicle (10 % DMSO in PBS) was administered by i.p. injection 30 min before MCAO in Sham, I/R, and EA + I/R groups. The 2ME2 dose (16 mg/kg) was consistent with previously published in vivo doses (Zhou et al. 2008). In experiment III, MW167 was dissolved in PBS with 10 % DMSO and used at a concentration of 1 mM (Jurynczyk et al. 2005), which was administered by i.c.v. injection 30 min before MCAO in the EA + MW167 + I/R group. The same volume of vehicle(10 % DMSO in PBS) was administered by i.c.v. injection 30 min before MCAO in I/R, EA + I/R, and EA + 2ME2 + I/R groups. The i.c.v. injection was performed as described previously (Zhao et al. 2012). 2ME2 was dissolved in PBS with 10 % dimethyl sulfoxide (DMSO) and administered by i.p. injection 30 min before MCAO in EA + 2ME2 + I/R group. The same volume of vehicle (10 % DMSO in PBS) was administered by i.p. injection 30 min before MCAO in I/R, EA + I/R, and EA + MW167 + I/R groups. The stereotactic coordinates were as follows: A, −1.0; R, 1.5; and H, 3.8 (coordinates corresponding to the right lateral ventricle) (Paxinos et al. 1980).

### Transient Focal Cerebral Ischemia

Animal Care: The animals were provided by the Experimental Animal Center of the Fourth Military Medical University (Xi’an, China). Male Sprague–Dawley (SD) rats weighing 260–300 g were housed in a specific pathogen-free environment with free access to sterile laboratory pellets and water. The experimental protocol was approved by the Ethics Committee for Animal Experimentation of the Fourth Military Medical University and was conducted according to the Guidelines for Animal Experimentation of the Fourth Military Medical University.

Focal cerebral ischemia was induced by a transient right middle cerebral artery occlusion (MCAO) in rats as described previously (Wang et al. 2012 b). Animals were anesthetized by intraperitoneal injection of 40 mg/kg SP for all surgical procedures. The regional cerebral blood flow (rCBF) was monitored using a disposable microtip fiber optic probe (diameter, 0.5 mm) connected through a master probe to a computerized laser Doppler main unit (PeriFlux 5000, Perimed AB, Sweden). Rats retaining >20 % of baseline perfusion during ischemia were excluded. Reperfusion was accomplished by withdrawing the suture after 120 min of ischemia, followed by suturing of the surgical wounds. The temporalis muscle temperature was monitored and maintained at 37.0–37.5 °C by surface heating or cooling during surgery until the rats recovered from anesthesia.

### Real-Time PCR

The ischemic penumbras of the cerebral cortex were harvested as described previously (Wei et al. 2014). PCR was performed under the following thermal cycling conditions: one cycle at 94 °C for 5 min; 25 cycles at 94 °C for 30 s, 55 °C for 60 s, and 72 °C for 30 s and one cycle at 72 °C for 10 min. Melt-curve analysis was used to identify different reaction products, including nonspecific products. The following primers for RT-PCR were designed by the TaKaRa corporation: HIF-1α (Fwd: CCA GAT TCA AGA TCA GCC AGC A, Rev: GCT GTC CAC ATC AAA GCA GTA CTC A), HO-1 (Fwd: AGG TGC ACA TCC GTG CAG AG, Rev: CTT CCA GGG CCG TAT AGA TAT GGT A), and GAPDH (Fwd: GGC ACA GTC AAG GCT GAG AAT G, Rev: ATG GTG GTG AAG ACG CCA GTA). Each sample was tested in triplicate. Samples were obtained from 3 independent experiments and analyzed for relative gene expression data using the 2^−ΔΔCT^ method.

### Western Blot

At 24 h after the last EA pretreatment and at 2, 6, 24, 48, and 72 h after reperfusion, HIF-1α expression in the ischemic penumbra of rats in Experiment I was evaluated. At 24 h after reperfusion, the Bcl2 and Bax expression in the ischemic penumbra of rats in Experiment II was evaluated. At 24 h after reperfusion, NICD and HIF-1α expression was measured in the rats in Experiment III as described previously (Deng et al. 2013, 2014). The following primary antibodies were used: mouse anti-HIF-1α (1:400, Novus Biologicals), mouse anti-Bcl2 (1:200, Aobo), mouse anti-Bax (1:200, Aobo), rabbit anti-Notch1 NICD (1:400; Abcam), and mouse anti-beta-actin antibody (1:1000, Abcam). Secondary horseradish peroxidase (HRP)–conjugated goat–anti-rabbit antibody or goat–anti-mouse antibody (Pierce Biotechnology Inc; 1:5000 dilution) was used. Changes in relative protein expression were represented as the ratio of the integrated optical density of the protein bands to that of β-actin. Quantitative analysis of the protein bands was performed using an Image-Quant 5.0 GE Healthcare Densitometer (GE Healthcare, Sunnyvale, CA). The experiments were performed independently in triplicate.

### Double Immunofluorescence Staining

HIF-1α/NeuN double immunofluorescence analyses were performed in the Sham, CON, and EA groups. Monoclonal mouse anti-HIF-1α (1:200, Novus Biologicals) and polyclonal rabbit anti-NeuN (1:1000, Millipore) were used. HIF-1α/Notch1 NICD double immunofluorescence analyses of the CON and EA groups were then performed. The secondary antibody used was FITC-labeled goat anti-mouse IgG (1:2000; Molecular Probes, USA) and Alexa Fluor 594-conjugated anti-rabbit IgG (1:1 000; Molecular Probes, USA). Finally, sections were observed, and images were captured using an Olympus BX-60 fluorescence microscope (Olympus Corporation, Shinjuku, Tokyo, Japan) with software named QCapture Pro.

### TUNEL Staining

At 24 h after ischemia/reperfusion, TUNEL staining of Sham, I/R, EA + I/R, 2ME2 + I/R, EA + 2ME2 + I/R groups from Experiment II was performed using an In Situ Cell Death Detection Kit (Roche Diagnostics, Mannheim, Germany) as described previously(Wang et al. 2013) and according to the manufacturer’s instructions. Images were viewed using an Olympus BX-60 fluorescence microscope. Integrated optical density/area of positive TUNEL staining in each group was measured by 2 blinded investigators using Image-Pro plus 5.1 software (Media Cybernetics, Inc., Bethesda, MD, USA).

### Neurobehavioral Evaluation and Infarct Volume Assessment

At 72 h after reperfusion, a neurological assessment of the rats in Sham, I/R, EA + I/R, 2ME2 + I/R, EA + 2ME2 + I/R groups was performed by a blinded observer using the 18-point scoring system reported by Garcia et al. (1995). The system consisted of the following six tests: (1) spontaneous activity, (2) symmetry in the movement of four limbs, (3) forepaw outstretching, (4) climbing, (5) body proprioception, and (6) response to vibrissae touch. The score given to each rat at the completion of the evaluation was the summation of all six individual test scores. Minimum neurologic score was 3; maximum was 18.

After neurological evaluation, rats were decapitated, and 2-mm thick coronal sections throughout the brain were stained with 2 % 2,3,5-triphenyltetrazolium chloride (TTC) to evaluate the infarct volume as described previously(Zhou et al. 2013). The areas of injured (white) and uninjured (red) were measured using an image analysis system (Adobe Photoshop 8.0, Adobe Systems Incorporated, San Jose, CA) for each slice. Infarct volume was calculated by Swanson and Sharp’s method to correct for edema: 100× (contralateral hemisphere volume—nonlesioned ipsilateral hemisphere volume)/contralateral hemisphere volume (Swanson and Sharp 1994).

### Statistical Analysis

SPSS 11.0 for Windows (SPSS Inc., Chicago, IL) was used. The HIF-1α and HO-1 mRNA levels, HIF-1α, Bcl2, Bax and NICD levels and infarct volumes were analyzed using one-way analysis of variance, and differences between the groups were detected using the post hoc Student–Newman–Keuls test. Each neurological deficit score was expressed as a median (range) and analyzed using the Kruskal–Wallis test followed by Dunn’s post hoc test. Other values were reported as the mean ± standard error of the mean (SEM) and analyzed among groups using one-way analysis of variance. When indicated by a significant *F* ratio, post hoc testing was performed using Scheffe’s test. Values of *p* < 0.05 were considered statistically significant.

## Results

### EA Pretreatment Significantly Enhanced HIF-1α and HO-1 Expression in the Ischemic Penumbra at the Early Stage of Reperfusion

**Fig. 1:**
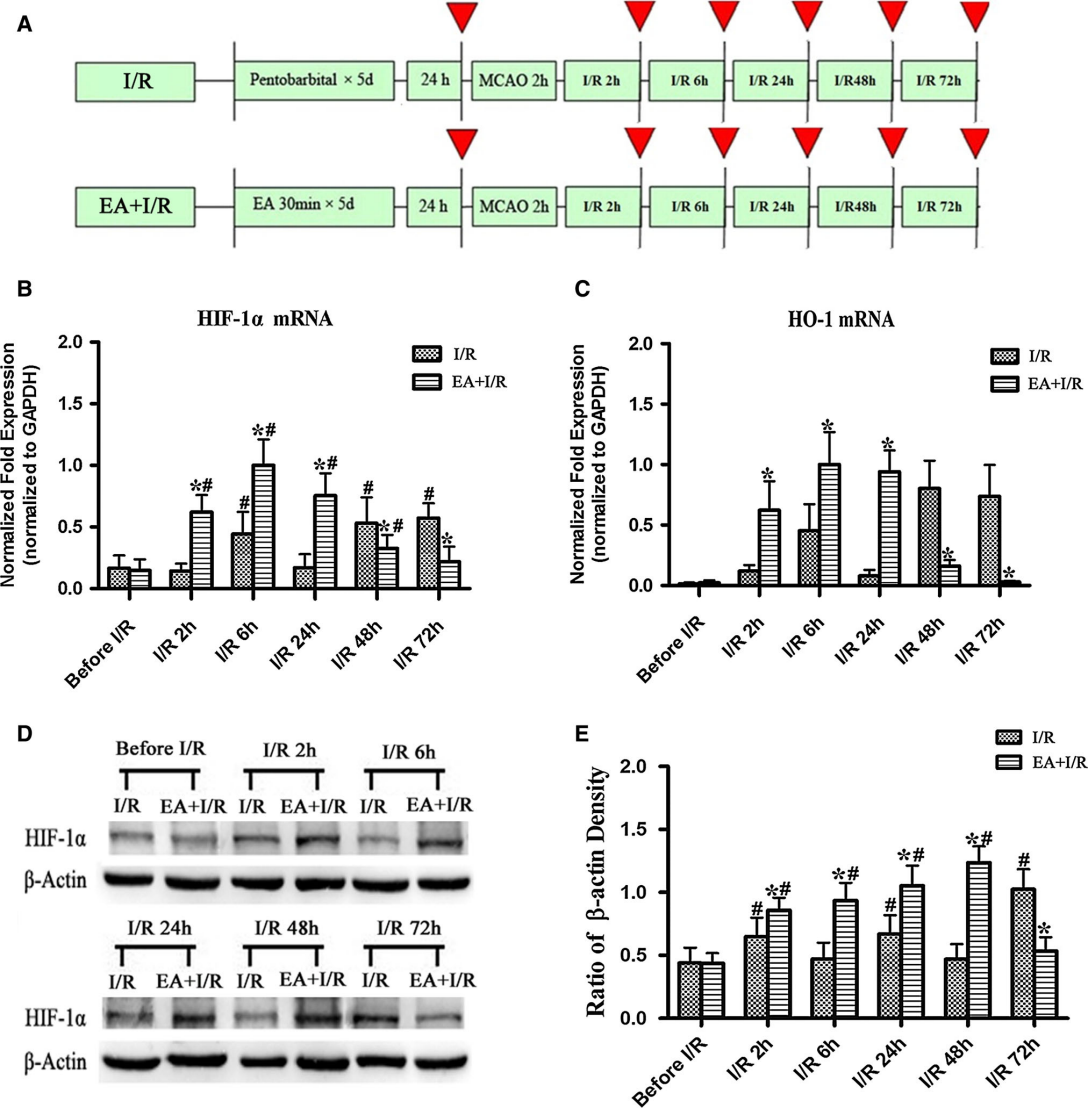
The expression of HIF-1α and HO-1 in the ischemic penumbra at 24 h after the final EA pretreatment and at 2, 6, 24, 48, and 72 h after ischemia/reperfusion (*n* = 8). **a** Experimental protocol. *Red arrows* indicate the time points at which the RT-PCR and Western blot analyses of HIF-1α or the RT-PCR analyses of HO-1 were performed. **b** RT-PCR analysis of the HIF-1α mRNA levels in the ischemic penumbra. **c** RT-PCR analysis of the HO-1 mRNA levels in the ischemic penumbra. **d** Representative Western blot bands showing HIF-1α expression in rats between I/R and EA + I/R groups. **e** *Bar graph* showing quantification of the Western blot analysis comparing the HIF-1α protein with β-actin (*p < 0.05 vs. I/R; #*p* < 0.05 vs. before I/R)

Expression of the HIF-1α and HO-1 genes in the ischemic penumbra was examined at the early stage of reperfusion. HO-1 is one of the downstream genes regulated by HIF-1α. The HIF-1α and HO-1 mRNA expression levels were significantly higher in EA + I/R group than in I/R group at 2, 6, and 24 h after reperfusion (*p* < 0.05). However, the expression levels of HIF-1α and HO-1 mRNA were significantly lower in EA + I/R group than in I/R group at 48 and 72 h after reperfusion (*p* < 0.05) (Fig. 1b/c).

Semi-quantitative Western blot analysis indicated that HIF-1α was significantly elevated at 2 h after reperfusion in EA + I/R group (*p* < 0.05 vs. before I/R) and reached the maximum level at 48 h after reperfusion. Furthermore, the level of HIF-1α in the EA + I/R group was significantly higher than in I/R group (*p* < 0.05) at 2, 6, 24, or 48 h (Fig. 1d, e).

**Fig. 2:**
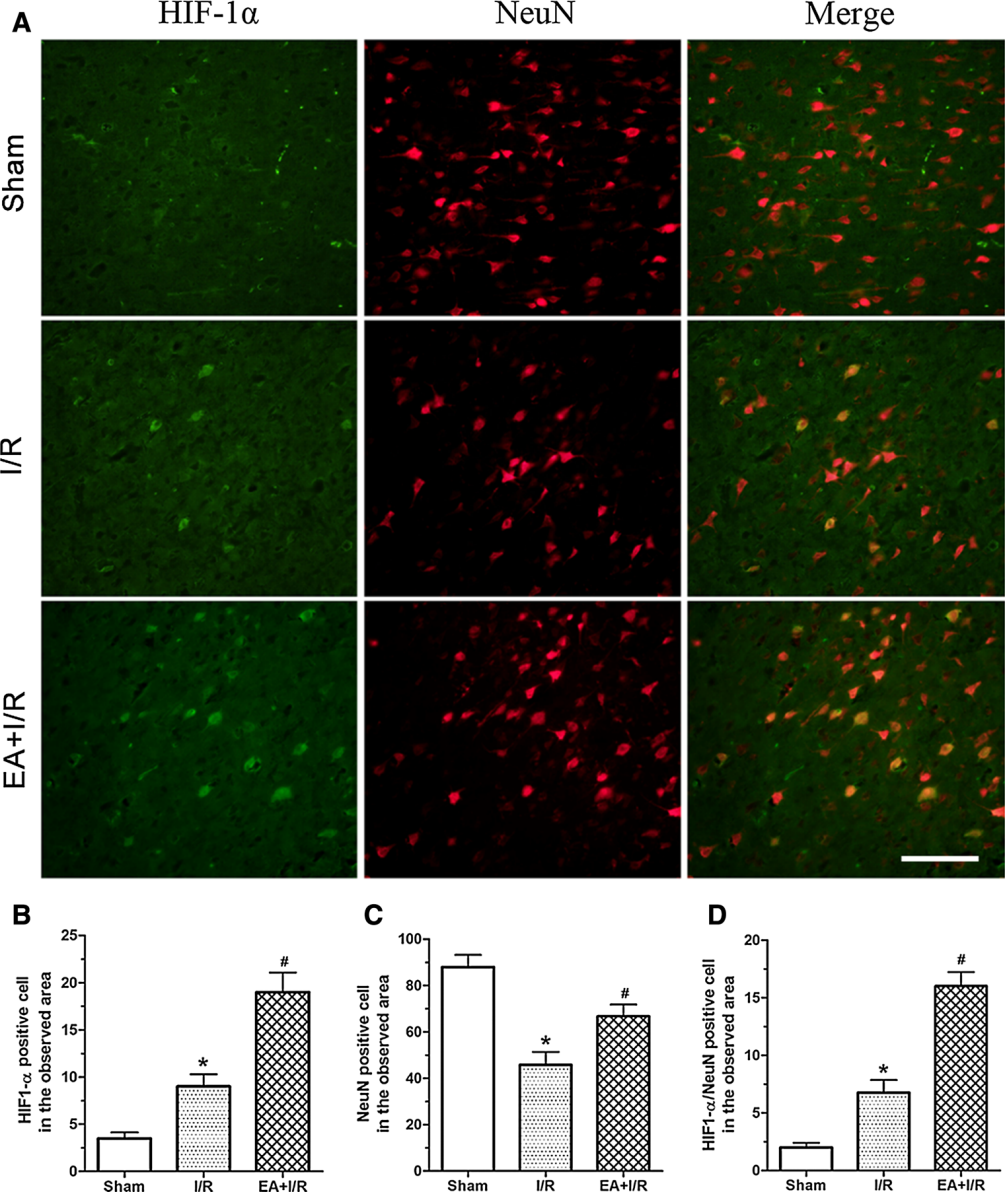
Immunofluorescence staining detected the expression of HIF-1α in neurons in the ischemic penumbra at 24 h after reperfusion. **a** Representative double immunofluorescence staining (*yellow*) of HIF-1α-positive cells (*green*) and NeuN-positive cells (*red*) in brain sections was displayed. *Scale bars* 100 μm. **b** Statistical analysis of the HIF-1α-positive cell numbers in the observed area. **c** Statistical analysis of the NenN-positive cell numbers in the observed area. **d** Statistical analysis of the HIF-1α/NenN double labeling cell numbers in the observed area (*p < 0.05 vs. Sham; #*p* < 0.05 vs. I/R)

We also evaluated whether neurons in the ischemic penumbra region expressed HIF-1α. The results indicated that there were more HIF-1α- and NeuN-positive cells in EA + I/R group than in I/R group 24 h after reperfusion. Furthermore, the number of HIF-1α/NeuN double-labeled neurons in EA + I/R group was significantly higher than in I/R group 24 h after reperfusion (Fig. 2). These results suggested that EA pretreatment increased the expression of HIF-1α and HO-1 in the ischemic penumbra at the early stage of reperfusion.

### HIF-1α Inhibitor 2ME2 Attenuated the Neuroprotective Effect of EA Pretreatment

**Fig. 3:**
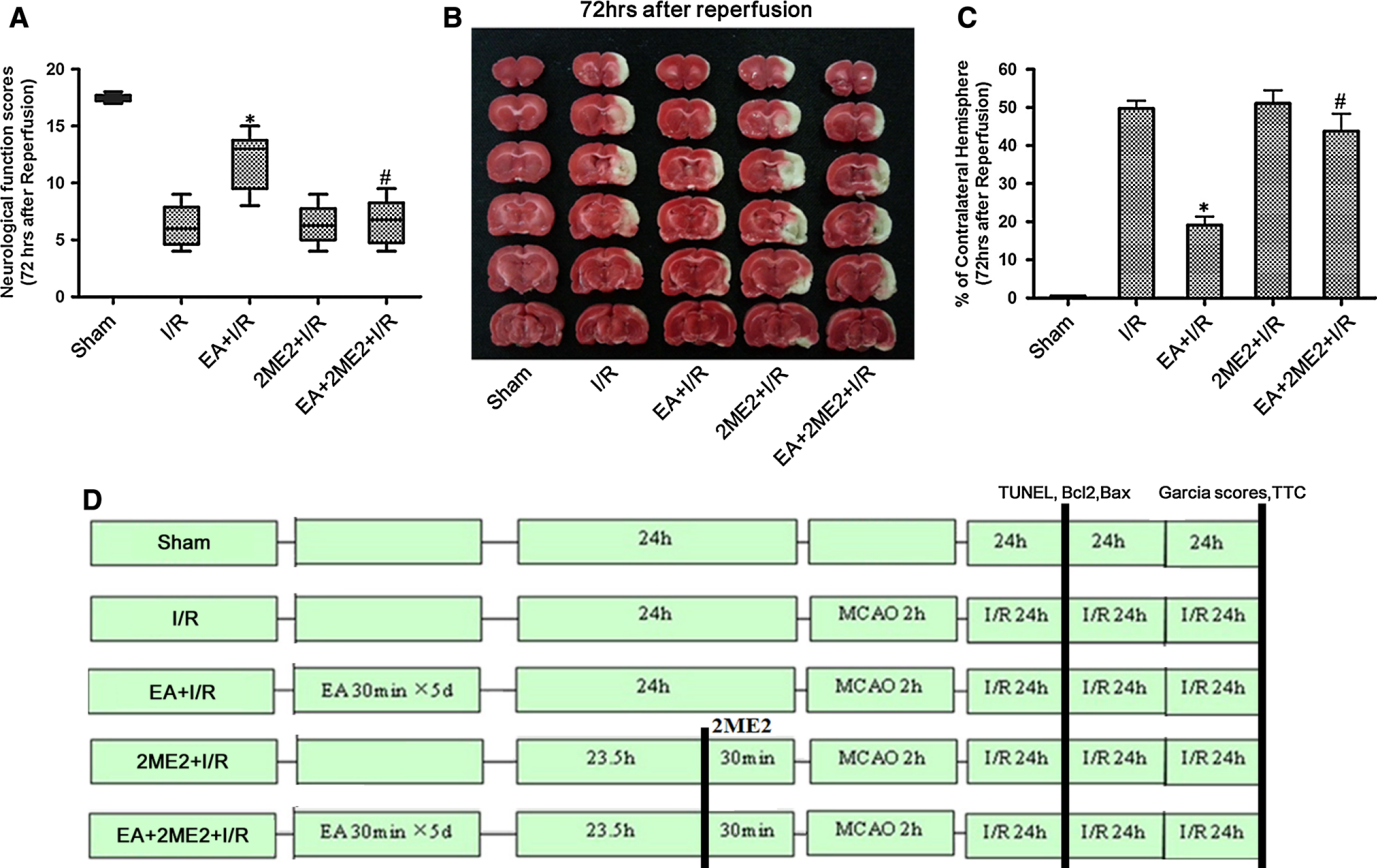
Neurological scores and infarct volumes at 72 h after reperfusion in the rats. **a** Garcia scores were tested at 72 h after reperfusion. **b** Representative brain infarct size indicated by TTC staining at 72 h after reperfusion. **c** Statistical analysis of the infarct size in every group (% of contralateral hemisphere) (*p < 0.05 vs. I/R; #*p* < 0.05 vs. EA + I/R). **d** Experimental protocol, SD rats were randomly divided into five groups (*n* = 8 each): Sham, I/R, 2ME2 + I/R, EA + I/R, and EA + 2ME2 + I/R group. At 30 min before MCAO, the 2ME2 were administered. At 72 h after ischemia/reperfusion, neurological function scores, and infarct volumes were assessed in every group. At 24 h after ischemia/reperfusion, TUNEL staining, the expression of Bcl2 and Bax was tested to evaluate neuronal apoptosis in every group

Neurological function was assessed using Garcia scores. At 72 h after reperfusion, rats receiving EA pretreatment showed significantly higher neurological function scores than those in I/R group (*p* < 0.05). However, the neurological function scores in the group given EA pretreatment with 2ME2 were significantly lower than in the group pretreated with EA alone (Fig. 3a).

At 72 h after reperfusion, the brain infarct volumes in EA + I/R group were significantly smaller than those in I/R group (*p* < 0.05). Interestingly, the infarct volumes in EA + 2ME2 + I/R group were significantly larger than those in EA + I/R group (*p* < 0.05). There was no difference between the I/R group and the group subjected to only 2ME2 (Fig. 3b, c). These results suggested that the neuroprotective effect induced by EA pretreatment can be alleviated by 2ME2 intervention.

**Fig. 4:**
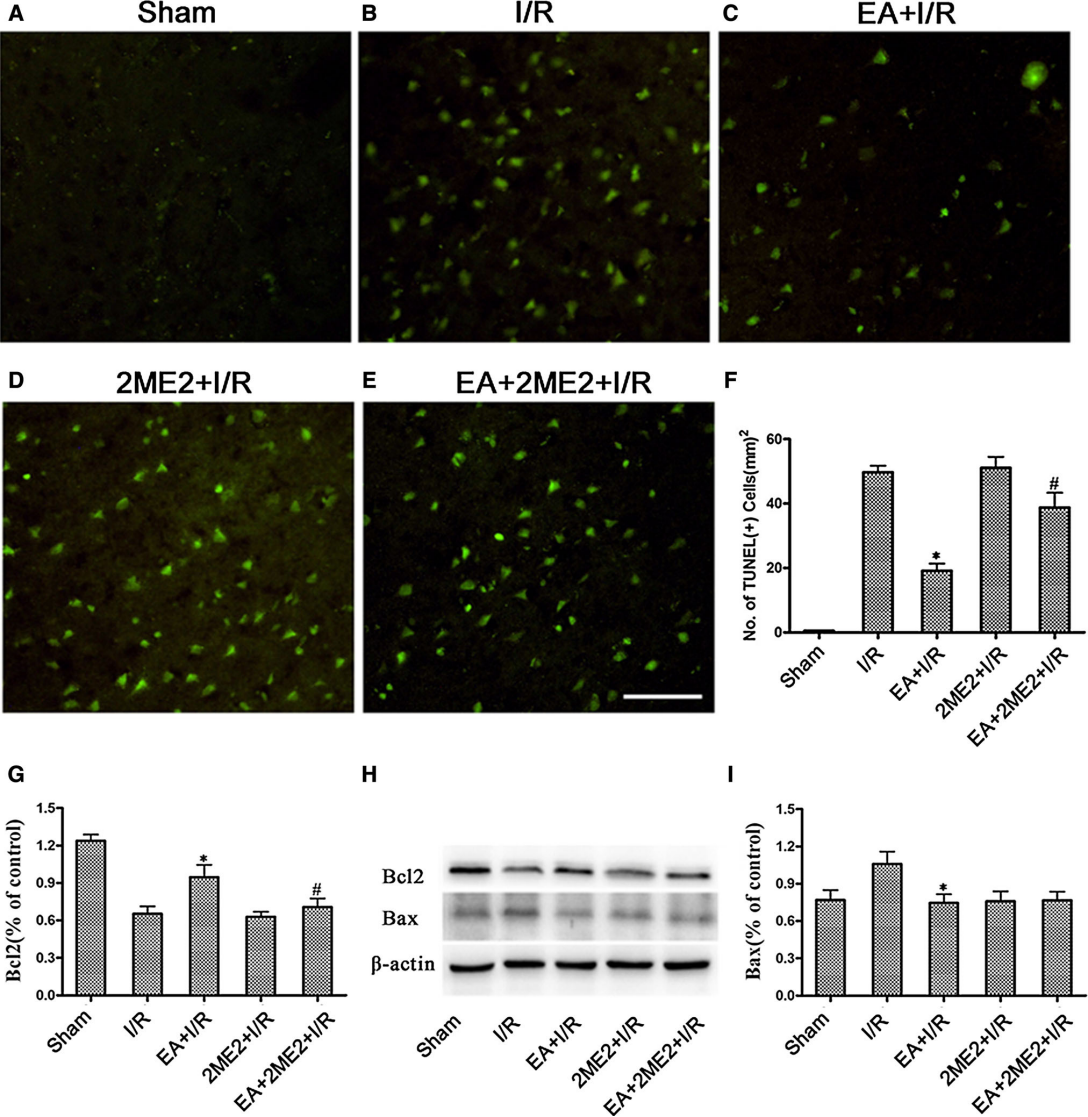
The representative TUNEL staining and the expression of Bcl-2 and Bax protein was tested at 24 h after reperfusion in every group. **a**–**e** The representative immunofluorescence staining of TUNEL-positive cells (*green*) in brain sections was displayed. *Scale bars* 100 μm. **f** Statistical analysis of the TUNEL-positive cells numbers. **h** Representative Western blot bands of Bcl-2 and Bax expressions at 24 h after reperfusion. **g**/**i** Statistical analysis comparing both Bcl-2 and Bax protein expression with β-actin (*p < 0.05 vs. I/R; #*p* < 0.05 vs. EA + I/R)

To assess the effect of 2ME2 on neuronal apoptosis, TUNEL staining on ischemic brain sections at 24 h after reperfusion was performed. There were more TUNEL-positive cells in the ischemic penumbras from the I/R and EA + 2ME2 + I/R groups than from the EA + I/R group (*p* < 0.05) 24 h after reperfusion (Fig. 4b, c, e). These results indicated that the EA pretreatment-induced reduction of neuronal apoptosis was attenuated by 2ME2.

We also assayed the expressions of the Bax and Bcl-2 proteins 24 h after reperfusion. As shown in Fig. 3g–i, rats only subjected to the EA pretreatment showed markedly up-regulated Bcl-2 levels (*p* < 0.05 vs. I/R) in the ischemic penumbra 24 h after reperfusion, whereas the Bcl-2 protein levels in the ischemic penumbra in EA + 2ME2 + I/R group were significantly lower than those in EA + I/R group (*p* < 0.05) (Fig. 4g/h/i). Meanwhile, the up-regulation of Bax in the ischemic penumbra was markedly reduced by the EA pretreatment (*p* < 0.05 vs. I/R).

### Interaction Between the Notch Signaling Pathway and HIF-1α

**Fig. 5:**
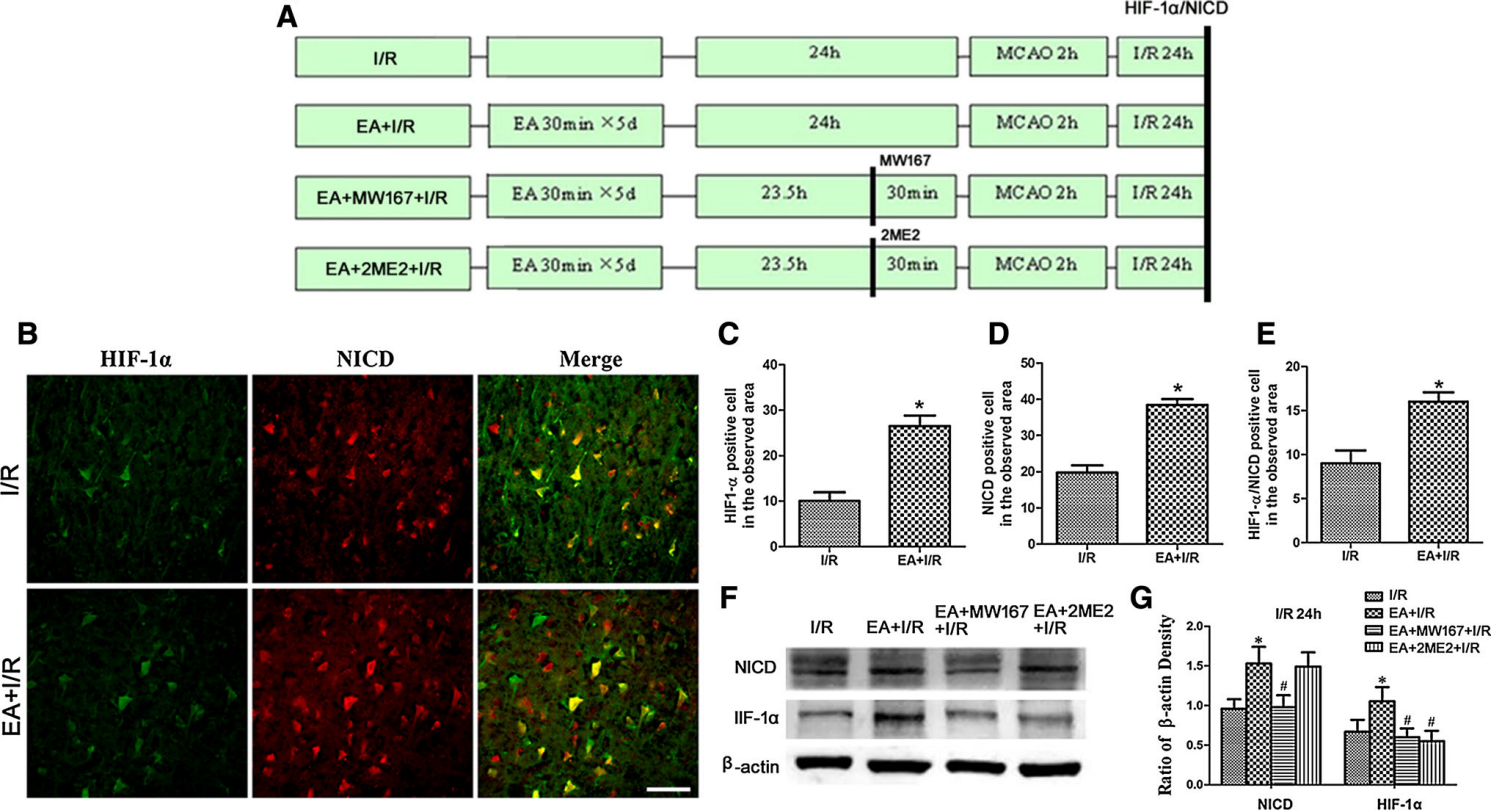
Representative double immunofluorescence staining and Western blot analyzing the expression of the HIF-1α and NICD at 24 h after reperfusion. **a** Experimental protocol. **b** Representative double immunofluorescence staining of HIF-1α-positive cells (*green*) and NICD-positive cells (*red*). *Scale bars* 50 μm. **c** Statistical analysis of the HIF-1α-positive cell numbers in the observed area. **d** Statistical analysis of the NICD-positive cell numbers in the observed area. **e** Statistical analysis of the HIF-1α/NICD double labeling cell numbers in the observed area (*p < 0.05 vs. I/R). **f** Representative Western blot bands of HIF-1α and Notch1 NICD expression in rats from the I/R, EA + I/R, EA + MW167 + I/R, and EA + 2ME2 + I/R groups at 24 h after reperfusion. **g** Statistical analysis comparing the expressions of the HIF-1α and NICD proteins with the expression of β-actin (*p < 0.05 vs. I/R; #*p* < 0.05 vs. EA + I/R)

As shown in Fig. 5b–e, there were significantly more HIF-1α- or NICD-positive cells in the ischemic penumbra in EA + I/R group than in I/R group (*p* < 0.05) 24 h after reperfusion. Furthermore, there were more HIF-1α/NICD double-labeled cells in the ischemic penumbra in EA + I/R group than in I/R group (p < 0.05) 24 h after reperfusion. These results indicated that HIF-1α and Notch1NICD were co-expressed in the ischemic penumbra.

To explore the relationship between the Notch signaling pathway and HIF-1α, we evaluated the expression of HIF-1α and NICD after the Notch signal was inhibited. As shown in Fig. 5f, g, the amount of HIF-1α and NICD in the ischemic penumbra of EA + I/R group was significantly higher than that in I/R group (*p* < 0.05) 24 h after reperfusion. However, MW167, which can inhibit the activation of the Notch signaling pathway, decreased the expressions of both HIF-1α and NICD (*p* < 0.05, EA + MW167 + I/R vs. EA + I/R). Furthermore, 2ME2 inhibited HIF-1α expression (*p* < 0.05, EA + 2ME2 + I/R vs. EA + I/R) but had no effect on the expression of NICD (*p* > 0.05, EA + 2ME2 + I/R vs. EA + I/R). These results suggested that inhibition of the Notch signal suppressed the expression of HIF-1α in the ischemic penumbra.

## Discussion

In the present study, we found that EA pretreatment significantly increased HIF-1α expression in the ischemic penumbra following reperfusion. In addition, immunofluorescence staining suggested that HIF-1α immunoreactivity was colocalized with NeuN immunoreactivity, indicating that the effect of EA pretreatment on HIF-1α expression may be neuron-specific. Baranova et al. reported that the neuron-specific inactivation of HIF-1α increased brain injury in a mouse model of transient focal cerebral ischemia (Baranova et al. 2007). A research showed that sevoflurane postconditioning protected the brain from focal cerebral ischemic reperfusion injury through up-regulating mRNA and protein expression of HIF-1α and its target gene, HO-1 (Ye et al. 2012). To address whether HIF-1α played a neuroprotective role in EA pretreatment after reperfusion, we inhibited HIF-1α expression using 2ME2, which selectively suppressed cellular HIF-1α protein synthesis without affecting HIF-1α mRNA transcription or the stability of the HIF-1α protein (Baranova et al. 2007). 2ME2 is a natural metabolite of estrogen that is known to inhibit HIF-1α in a dose-dependent manner (Ricker et al. 2004). The dose (16 mg/kg) of 2ME2 can effectively inhibit the expression of HIF-1α and its target gene, VEGF(Zhou et al. 2008; Zhu et al. 2014). The administration of 2ME2 attenuated the beneficial effects of EA on infarct volumes, neurological outcomes, and apoptosis. These results suggest that the up-regulation of HIF-1α may contribute to the neuroprotective effects of EA pretreatment against focal ischemia. Certainly, 2ME2 has other features, such as anti-tumor, anti-angiogenesis, anti-cytotoxicity, and anti-proliferation. These features may have some effect on our research.

Apoptosis is one of the major causes of neuronal injury following cerebral ischemia, and the inhibition of apoptosis reduces ischemic injury. HIF-1α is a transcription factor that plays a key role in regulating the adaptive response to hypoxia and is involved in regulating apoptosis under hypoxic conditions. Increasing HIF-1α protein stability reduces brain injury after transient cerebral ischemia (Kunze et al. 2012). We found that EA pretreatment significantly reduced the number of apoptotic neurons, up-regulated the anti-apoptotic protein Bcl-2 and down-regulated the pro-apoptotic protein of Bax in the ischemic penumbra, suggesting that EA pretreatment alleviates neuronal apoptosis. Yang et al. reported that Panaxynol protected cortical neurons from ischemia-like injury by up-regulation of HIF-1α expression and inhibition of apoptotic cascade (Yang et al. 2010). However, how HIF-1α exerts its neuroprotective effects after EA administered remains investigation.

It is still controversial whether the activated canonical Notch signaling is beneficial to the ischemic cerebral tissues. Many researchers have found that the activation of Notch signaling was involved in the ischemic tolerance induced by inhalation anesthetics preconditioning (Zhang et al. 2014; Yang et al. 2012). However, other reports was consistent with the opinion that Notch signaling can induce neuronal cell death (Arumugam et al. 2011). The discrepancies might be explained by the injury model, the severity of injury and the timepoint of notch inhibition. In our past work, we found that EA pretreatment-induced tolerance against focal cerebral ischemia through activation of the canonical Notch signaling pathway (Zhao et al. 2012). Notch signaling and the cellular hypoxic response have been shown to be functionally integrated (Gustafsson et al. 2005; Kamarehei and Yazdanparast 2014). Zheng et al. reported that FIH-1 played a role in mediating hypoxia-potentiated Notch signaling, suggesting that Notch ICD could sequester FIH-1 from HIF-1α, thus decreasing HIF-1α function (Zheng et al. 2008). Lee JH et al. found that the transcription of HIF-1α gene was up-regulated by the induction of Notch signaling under hypoxic conditions (Lee et al. 2009). Our results also indicated the inhibition of the Notch signal suppressed HIF-1α expression in the ischemic penumbra, whereas HIF-1α inhibition had no effect on NICD expression. These results suggest that the up-regulation of HIF-1α during the EA pretreatment is mediated by the activation of the Notch pathway. However, our results do not agree with a recent report by Cheng et al. showing that the NICD and HIF-1α collaborated to engage pro-inflammatory and apoptotic signaling pathways in stroke (Cheng et al. 2014). The discrepancies might be explained by the different levels of HIF-1α expression in the ischemic penumbra after focal cerebral ischemia, because HIF coordinated both cell survival and death mechanisms. The research of Cheng focused on the expression and function of NICD and HIF-1α in ischemic stroke, while our focus was the benefit of up-regulation of NICD and HIF-1α expression induced by EA pretreatment in the focal cerebral ischemic injury. Besides, the time of MCAO in Cheng’ research was much less than that in our research.

In summary, the current investigation indicates that EA pretreatment affords strong protection against transient cerebral ischemic injury and increases the protein expression and activation of HIF-1α in rats. The beneficial effects of EA pretreatment may be mediated by the activation of the Notch signaling pathway.
